# Incarcerated Femoral Hernia Repair with Ventralex™ Hernia Patch through Same Skin Incision and Suprainguinal Laparotomy

**DOI:** 10.1155/2018/9719310

**Published:** 2018-10-22

**Authors:** David Aranovich, Veacheslav Zilbermints, Oleg Kaminsky

**Affiliations:** ^1^Department of General Surgery, Beilinson Hospital, Rabin Medical Center, Petach Tikva, Israel; ^2^Sackler Medical School, Tel Aviv University, Tel Aviv, Israel

## Abstract

**Purpose:**

To report our experience with incarcerated femoral hernia procedure, which allows laparotomy through same inguinal skin incision, inspection and resection of compromised bowel, and preperitoneal tension-free transabdominal repair with Ventralex™ Hernia Patch.

**Materials and Methods:**

The suprainguinal laparotomy was performed via same groin incision without compromising iliopubic tract. The femoral ring was sealed with Ventralex™ Hernia Patch pulled through the abdominal cavity and secured outside. Five consecutive patients diagnosed with incarcerated femoral hernias were operated. All of them required laparotomy, either for bowel resection (*n* = 3) or for inspection of viability (*n* = 2).

**Results:**

All patients tolerated the procedure well. There were no wound or mesh infections, incisional hernias, or recurrences during follow-up.

**Conclusions:**

Our easy-to-master operative approach to incarcerated femoral hernia allows easy access to abdominal cavity through same groin incision without compromising iliopubic tract or midline laparotomy. Reduction of incarcerated bowel and its inspection and resection can be safely performed. The femoral ring defect can be effectively obliterated with Ventralex™ Hernia Patch.

## 1. Introduction

Femoral hernias are less frequently encountered in general surgical practice than other types of primary groin hernias. They comprise less than five percent of all inguinal hernias. Because of its anatomically complex surrounding, challenging repair options, and lack of experience, it has been coined “the bête noire of hernias” [1, 2]. Moreover, the surgeon is confronted by confusing choice of operative approaches named after very respectful masters of the past, such as Mercy, Henri, McEvedy, Lotheissen, and Fergusson [3]. Surgery for femoral hernia incarceration triggers even more frustration, because of tightly oriented vascular structures around the hernia gate, fear of iliopubic tract violation, need of laparotomy for adequate bowel inspection and/or resection, and finally the choice of repair.

Young surgeons fear femoral hernia not only because of their lack of experience with this type of a hernia but also because of numerous horror stories told them by their senior colleagues about this beast. Nevertheless, most of us approach incarcerated femoral hernia with some degree of apprehension.

We would like to describe incarcerated femoral hernia procedure, utilizing novel technique which allows small laparotomy through same skin incision, inspection and resection of compromised bowel, and durable tension-free transabdominal repair with Ventralex™ Hernia Patch.

## 2. Technique

**Figure 1:**
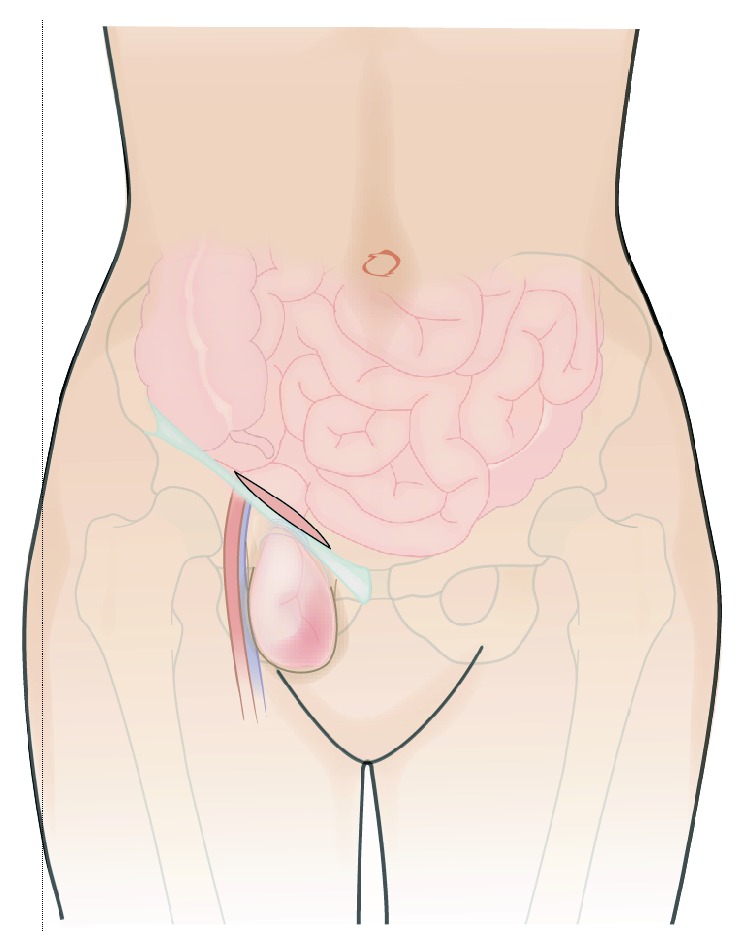
The skin incision is placed parallel and slightly above the inguinal ligament.

The patient is placed in supine position, and after induction of general anesthesia, the patient was prepped and draped in regular surgical fashion. The oblique incision is made slightly above the inguinal ligament and carried medially to the pubic tubercle (Figure 1). This incision placement allows easy access to the femoral hernia sac and upper thigh from below and to the right lower abdominal wall from above. The subcutaneous tissue is dissected; hernia sac is cleared, and hernia neck is exposed. The sac is incised, and its content is inspected.

**Figure 2:**
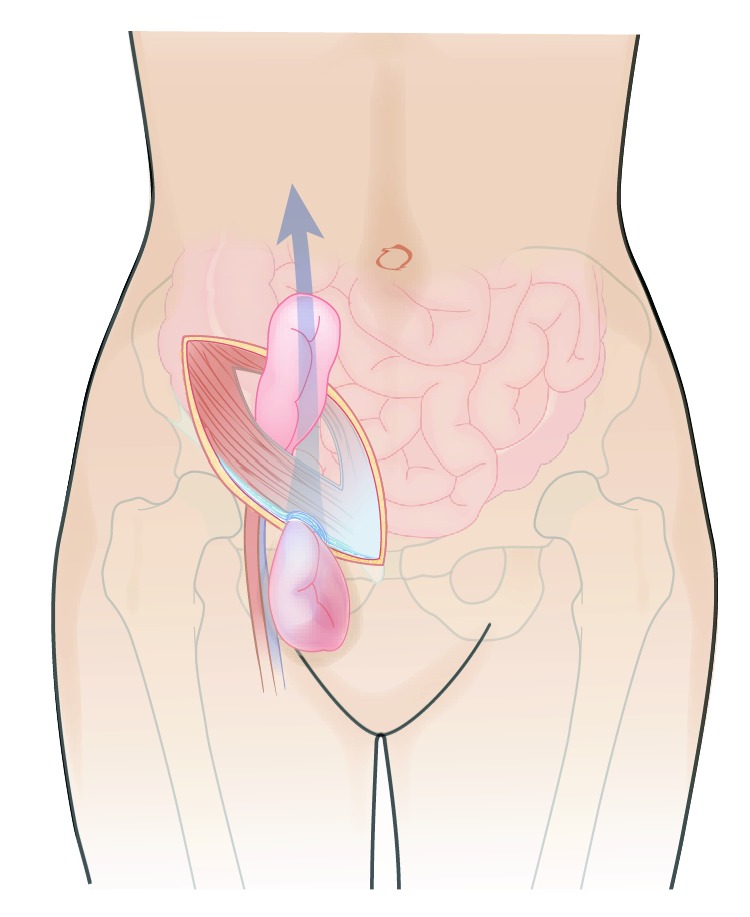
Incarcerated bowel loop is pulled through the femoral ring and eviscerated through the abdominal wall incision.

The procedure is performed only when laparotomy is needed for resection or viability assessment. The skin incision is undermined upwards to expose the aponeurosis of external oblique muscle 4–5 cm above inguinal ligament. The aponeurosis is divided transversely or obliquely above the inguinal canal. Muscle-splitting gridiron-type incision is made to enter the abdominal cavity. This approach allows 4–6 cm laparotomy incision. The incarcerated bowel is then pulled to the abdominal cavity and eviscerated for viability assessment (Figure 2).

We try to avoid violation of iliopubic tract during hernia gate incision. The femoral ring is released by incision of a small portion of inguinal ligament in the cranial direction and/or the lacunar ligament of Gimbernat, just to ensure appropriate relaxation of the hernia gate for the release of the strangulated bowel.

If the incarcerated bowel loop is undoubtedly nonviable, it may be resected with linear stapler first and then the stumps are pushed back to the abdominal cavity and retrieved through the laparotomy incision.

This suprainguinal laparotomy allows enough room for manipulation with incarcerated bowel, viability assessment, resection, anastomosis, and subsequent hernia repair.

**Figure 3:**
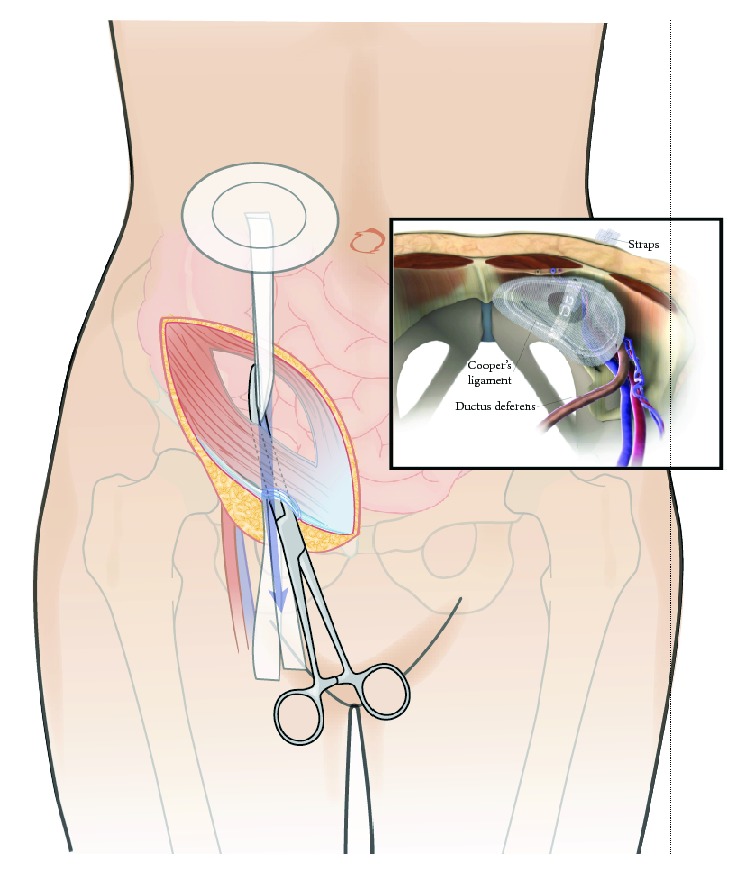
Ventralex™ Hernia Patch is pulled through the femoral ring and obliterates the entire myopectineal orifice from inside.

The repair of the femoral ring opening is performed from the abdominal cavity. Long Kelly clamp is passed from outside through the femoral canal to the abdominal cavity. Ventralex™ Hernia Patch straps are grasped and pulled through the femoral canal until the disc of the patch is tightly tugged against the pelvic wall. This maneuver not only allows complete obliteration of the hernia opening but also forms a wide intraperitoneal coverage of the entire myopectineal orifice of Fruchaud (Figure 3). The straps are trimmed outside the femoral ring and secured tightly by suturing one strap to the Copper's ligament and another to the inguinal ligament. As a final step of Ventralex patch placement, we pay close attention to ensure that the PTFE layer of the patch is properly spread out and secured in stable position without folds. Folding of the patch exposes its polypropylene part to the intra-abdominal content. This may result in many mesh-related complications, including adhesions, bowel obstruction, bowel erosion, and perforation.

The suprainguinal laparotomy is closed by approximation of muscle and fascia.

## 3. Results

The first author of this paper (DA) used this technique since 2007, in 5 consecutive patients, three males and two females. Short-term follow-up was performed at regular clinic visits up to three months postoperatively. Long-term follow-up was performed with the universal electronic database system at the time of manuscript preparation.

In three patients, incarcerated small bowel was reduced back to the abdominal cavity through the suprainguinal laparotomy and then resected. In one patient, ischemic small bowel was resected at the incarceration site with a linear stapler and its stumps are retrieved through the suprainguinal laparotomy and then anastomosed. In one patient, the incarcerated bowel regained its vitality after being released into the abdominal cavity. No recurrences, mesh, or wound infections were observed.

## 4. Discussion

Incarcerated femoral hernia poses several distinctive surgical challenges. Its treacherous anatomy is explained by intricacy of the femoral inlet, which creates a rigid circle, limited by the pubic crest and Cooper's ligament posteriorly, the inguinal ligament anteriorly, the lacunar ligament medially, and the femoral vein laterally. This tense environment around the hernia neck creates limited options for hernia gate incision and further exposure of the hernia content to allow safe inspection and resection of the incarcerated bowel. To overcome these obstacles, several techniques are generally used. Wide incision of the iliopubic tract can achieve a good exposure for bowel inspection and resection. But this method creates a large defect in the myopectineal orifice with inguinal floor violation, which is rather difficult to repair because of the flimsy tissues in the groin area and loss of natural support of intact iliopubic tract. If the bowel viability is put in question or resection is required, midline laparotomy or laparoscopy is generally employed for viability assessment and resection of the bowel. Emergency surgery for incarcerated femoral hernia has been associated with up to 25% complication rate [4]. The most popular technique for femoral hernia repair is plug mesh repair first introduced by Irving Lichtenstein [5]. This method is widely used due to its simplicity and reproducibility and because it is generally safe [4–7]. Major complications of plug mesh repair, such as mesh migration, bowel obstruction, bowel erosion, and recurrence, have been reported [8–10]. Whereas plug mesh repair is a good technique for elective femoral hernia cases with small myofascial defects, it may be less suitable for many emergency strangulated cases with large hernia defects which often call for more sizable prosthetic tension-free solution for durable and safe repair.

The Ventralex™ Hernia Patch is a self-expanding polypropylene and EPTFE (expanded polytetrafluoroethylene) patch that allows for an intra-abdominal retrofascial placement through relatively small incisions without the need of extensive lateral dissection. These composite polypropylene/EPTFE hernia patches have been proved to be an effective solution for small umbilical, epigastric, and ventral hernias with good long-term results and low recurrence rates [11–13].

In this article, we describe another option to approach incarcerated femoral hernia, utilizing one skin incision for both hernia sac exploration, suprainguinal laparotomy for bowel viability assessment, and/or resection. We perform limited incision of the hernia gate just to allow its relaxation for relief of strangulation and content inspection. Furthermore, Ventralex™ Hernia Patch pulled from within the abdominal cavity and lodges in an extremely favorable preperitoneal position obliterating the entire myopectineal orifice. We believe that this procedure offers a simple option for dealing with incarcerated femoral hernias.
